# Peer communication on sex and sexual health among youths: a case of Debre Berhan university, Ethiopia

**DOI:** 10.11604/pamj.supp.2016.25.2.9631

**Published:** 2016-11-26

**Authors:** Takele Gezahegn, Zewdie Birhanu, Mamusha Aman, Muluken Dessalegn, Asmamaw Abera, Josephat Nyagero

**Affiliations:** 1 Department of Public Health, Institute of Medicine and Health Science, Debre Birhan University, Debre Birhan, Ethiopia; 2 Department of Health Education and Behavioral Sciences, College of Public Health and Medical Sciences, Jimma University, Jimma, Ethiopia; 3 Amref Health Africa in Ethiopia, Ethiopia; 4 Amref Health Africa, HQ, Kenya

**Keywords:** Debre Berhan university, peer communication, sexual health talk, grounded theory

## Abstract

**Introduction:**

Friends are considered an important source of advice and information about sex. Conversations about sex among young people tend to generate norms that influence positive or negative pressure on individuals to conform to group standards. The aim of the study was to explore peer communication on sex and sexual health.

**Methods:**

Grounded theory qualitative study design was employed using focus group discussions and participant observation. Participants were selected using criterion purposive sampling. Semi-structured guides and checklists were used as data collection tools. Information was audio-recorded and transcribed verbatim and uploaded to ATLAS.ti 7 software for coding. Data collection and analysis were undertaken simultaneously using constant comparative analysis.

**Results:**

Students talked with peers and sexual partners about sex more than sexual health issues. Common places of talk included dormitory, begtera (near dorm where students meet), and space (reading rooms). Whereas, time of talk, either in a group or with just their close friends or sex partners, included during training, evening and weekend time, during walking together, and break time. Students used verbal and non-verbal and formal and informal communication styles.

**Conclusion:**

The content, place, and time for discussions about sex were influenced by gender, social-cultural norms (e.g. religion), rural vs urban living, and the occurrence of sexual health issues (e.g, sexually-transmitted infections or unwanted pregnancies). Priority should be given to designing audience-specific strategies and messages to promote discussions about sex and to encourage safe sexual practices. Primary target groups should include female and rural students, who are predisposed to risky sexual behavior.

## Introduction

Sexuality is a central aspect of being human throughout life; it encompasses sex, gender identities and roles, sexual orientation, eroticism, pleasure, intimacy and reproduction. It is experienced and expressed in thoughts, fantasies, desires, beliefs, attitudes, values, behaviors, practices, roles and relationships but not all of them are always experienced or expressed [1]. Friends are considered an important source of advice and information about sex. Young women and gay men usually find it easier than heterosexual males to talk seriously and openly with their friends. Embarrassment, lack of trust and concern about not being taken seriously inhibit young heterosexual men from disclosing private information to friends. Young people are more likely to talk openly with close friends they can trust and who will take the topic seriously [2]. The sexuality of young people is, to a large extent, shaped and influenced by conversations and interactions with peers [3], for example the perception to homosexuality and use of condoms.

In many contexts young people get conflicting and confusing messages about sexuality and gender due to silence about the topic, disapproval of open discussion of sexual matters by adults and embarrassment to talk about sexuality [4]. Sexual communication has been noted in various situations to be predictive of condom use. Among incarcerated Latino adolescents with high numbers of sexual partners in the United State of America (USA), it was reported that youth who communicated with their sex partners about each other’s sexual history were significantly more likely to use condoms. Sexual communication has also been reported as a means to self-efficacy among heterosexuals in Holland [5]. As a result of a cultural taboo, adolescents in many developing countries rarely discuss sexual matters explicitly with their parents. Most information for their limited knowledge often comes from peers of the same sex, who may themselves be uninformed or incorrectly informed [6]. Men and women still seem to speak different languages when it comes to sex, but lack of communication affects behaviors and attitudes [7].

Considering the risk of contracting human immune-deficiency virus/acquired immune-deficiency syndrome (HIV and AIDS) and the increased spread of dangerous sexually transmitted infections (STI), talking to partners before having sex for the first time could save life. A good sexual relationship takes work and good communication which is one of the biggest problems for every couple. If someone decide to practice safer sex, the best time to have this talk with partners is before having sex [8].

The importance of discussing sexual health, together with the difficulties of doing so, are increasingly recognized [2]. Adolescents often lack basic reproductive health information, skills in negotiating sexual relationships and access to affordable confidential reproductive health services. Many adolescent lack strong and stable relationships with their parents or other adults to get reliable information about their reproductive health concerns, which puts them at risk for various reproductive health challenges [9].

Most studies examining young people`s sexual health-related discussions have focused on their communication within and about healthcare service provision situations. Parent-child communication was well studied but to date, there is little empirical and theoretical literature examining young people`s sexual health talks with their peers regarding their sexuality and sexual health [7–10].

Therefore, the objectives of this study was to explore the social contexts, contents and factors influencing peer communication on sex and sexual health among Debra Birhan University students. The result can be used to guide communication strategies, counseling, peer-to-peer sex education and training to enhance sexual life of young people.

## Methods

### Study setting and participants

A grounded theory qualitative study design was used to explore how peer communicate about sex and sexual health among youth from Debre Birhan University during March to April, 2014. Grounded theory is one of the commonly used among the qualitative study design (It is a way of thinking and studying social phenomena and it provides techniques and procedures for gathering and analyzing data aims to generate a theory explicitly from data). The University is located in Debre Birhan town in North Shewa, Amhara regional state, which is 130 Km from Addis Ababa. The University had a total of 14,812 studnts:10,006 regular (full boarding at the University), 6,596 male and 3387 female students [11]. Participants for the study were selected from regular students to gather primary data. The inclusion criteria were: being aged 18 years or older, unmarried, registered as a regular student regardless of department and consenting to participate. Those students who are critically sick during the data collection period were excluded from the study. In total, 69 students (37 male and 32 female) were participated in the study. The mean age of participants was 21.2 years with minimum of 19 and maximum 25. Sixty three were living in the dormitories whereas 32 of the students were from rural areas.

### Sample size and sampling technique

Eight focus group discussions (FGDs) based on saturation of information of 7 to 11 participant, structured by sex (37 male and 32 female) and year of study but varied by other variables were conducted. Sample size was determined by saturation of data and categories that reached after no new themes were identified based on iteration level and constant comparative analysis (CCA) and criterion purposive sampling was used.

### Data collection method and tools

The data was collected through FGDs and participant observation. The sampling and FGD process was flexible and continued until theoretical saturation. The moderator and the participant observer was the principal investigator (PI) for all FGDs and assistant moderator was gender matched to participants. The discussions were prescheduled and took place in class rooms. Such exclusion guaranteed optimum privacy. Each FGD lasted 60 to 80 minutes to conduct. Participants were observed in different places in the campus. The researcher attended and participated in the activities the youth performed and socialized with them for a total of 20 days. He listened to what the students talked about sex and sexual health issues with their peers or sex partners.

Relevant field notes were taken using checklist and unclear ideas were supported by findings from steering probes added on the FGD guide. Data collection tools included FGD guide and observation checklist. Observation checklist was used in order to capture what and how the students discussed.

The guide was flexible and semi-structured open-ended questions emerged from the study objectives and adapted from literatures. Each English version FGD guide was translated into Amharic which is the common language, and vice versa. Voice recorder was used to record FDGs in addition to notes taken during discussion. The last form of data was taken by keeping a reflective way that allowed the researchers to describe their feelings and added rigor to this qualitative inquiry as the investigators were able to record their reactions, assumptions, expectations, and biases about the research process.

### Data management and analysis procedures

Data was organized, reduced through summarization and categorization, and patterns and themes in the data were identified and linked during analysis. Focus group discussions were transcribed verbatim. All audio taped and field notes of the discussions were fully transcribed to Amharic then translated into English language. Finally, the data were analyzed using grounded theory constant comparison approach based on Strauss and Corbin`s recommendation. To manage the overall coding and memo developing process ATLAS.ti 7 Software was used.

The codes were assigned to the data and compared with the data from other participants in relation to their underlying meanings, patterns, occurrences and similarities in sexual talk. Codes were emerged to families (categories) and super-families (themes). Throughout the process memos were written to elaborate the categories. First, 210 codes emerged and categorized under 20 sub-themes and aggregated to 5 themes through open, axial and selective coding process. All those codes used were inductive and categories were formed by clustering similar codes and giving them name. Six major themes, divided into two thematic sections (one central theme and five themes) were identified. These are: Social Contexts of talk, Contents of Talk, Means/Channels of Communication, Functions of talk, and Socio-cultural Influences. The central theme was talking about Sex and Sexual Health (Table 1).

**Table 1:** Matrix table based on the study themes (central theme, sub-themes and categories) identified from the data

Talk about Sex and Sexual Health (Central Theme)					
Super Families (Themes)	Social Contexts of Talk	Contents of Talk	Means/Channels of Communication	Functions of talk	Socio-cultural Influences
Families (Categories)	-Place (Where) of Talk -Time (When) of Talk -How to talk -Partners (With Whom) to Talk	-Sex and sexual relationship Love and relationship, -HIV/AIDS issues, STIs, -Condom and contraceptive utilization, -Problem occurred, -Pregnancy, abortion, Cohabitation, negotiating female and Previous sexual life	-Verbal and non-verbal “sign languages” -Formal and informal Telephone -Social-media (face book or internet) Agent	-Advantages of Talk -Disadvantages of Talk	-Male-Female Differences -Urban-Rural Differences -Religion and Religious teaching -Culture and Globalization

**Figure 1:**
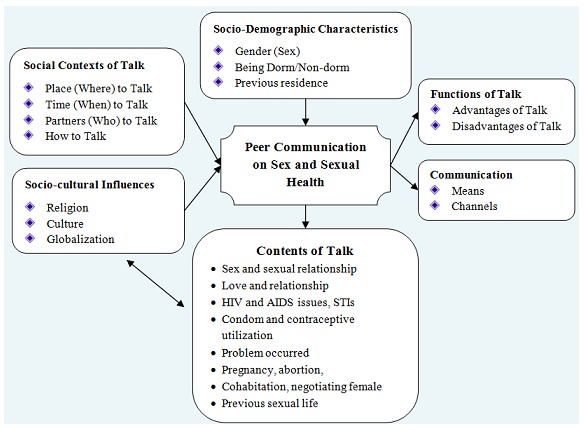
Theoretical frame work brought up by the study data, Debre Berhan university, may 2014 (Takele et al)

Finally, the following theoretical framework was developed from the data obtained from FGDs discussants by the researcher considering the research objective and topic. Among the socio-demographic variables sex and being dorm or non-dorm affects the sexual talk and behavior of the students. The social contexts (where, when, with whom and how) of the talk also affects sexual talk among students. In the same way socio-cultural factors (culture, religion and globalization) are the major influence of the sexual talk either inhibit or enhance sexual talk as well as the sexual practices among the students. Contents of talk are as the result of sexual talk among the students. Functions of talk (its advantages and disadvantages) and the means or channels of communication are related to sexual talk among the students (Figure 1). Several strategies were used to maintain the trustworthiness of the study. Developing an early familiarity with culture of participants to build trust and rapport, and interactive questioning was employed. Focus group discussions findings were supported by findings from participant observation, and the researcher gets back to participants to see whether the transcribed data correctly represent their points of views for participant validation (Figure 1).

### Ethical consideration

Ethical clearance was obtained from the Research and Ethical Committee of the college of Public Health and Medical Sciences, Jimma University. A formal letter was obtained from the department of Health Education and Behavioral Sciences and Debre Berhan University. All participants were given detail information about the purpose of the study, no names were used to report findings and informed verbal consent was obtained from all participants for their willingness to participate in the study.

## Results

### Talking about sex and sexual health

Talking on sex was most common than talking on other sexual health issues even though the benefit of talking on different topics of Sexual and Reproductive Health (SRH) was emphasized by the FGDs discussants. A 21 years old 3rd year male student from History Department said: *“When there is a talk … mostly the talk is about sexual intercourse. … Mostly the talk is sex talk, for example talk on how to meet or get partner and practice sex.”*

The talk about sex typically consisted of descriptions about how they have sex, where they have sex, whom they had sex with and what sex acts they engaged in. Male and female students discuss also on how they make relationship with opposite sex.

Focus group discussants also reported that male students talk on how to negotiate with female students for sexual activities. Students expressed talking about sex as an entertainment for them and unlike talking on other issues except for few religious and conservative students. In between their talks, students play about their relationship like how they stay in love and relationship about other topics of SRH. Talking on SRH issues among students was uncommon except few students talking on the issues like HIV, contraception, and diseases like STIs. Such talk was observed between close friends or sex partners as 23 years old 3rd year female student from Civics Department said: *“…partners rise during this time different sexual and reproductive health issues like… HIV, contraception, diseases cases, STIs and etc.”*

Participant observations also revealed that male and female walking and talking together on the way from campus to the town bars and restaurants. Talks among few friends on topics like HIV and AIDS, VCT, STIs, unwanted pregnancy and contraceptive use including condom utilization were observed. They could help a lot in making the sexual practices among the students healthier. However, students consider using condom as the role of male and using contraceptives as the responsibility of females.

### Social contexts of talk

From the FGDs, the discussants gave their response for the place, time, with whom, and how students communicate about sex and sexual health issues.

#### Place (where) of talk

Places on which students talk includes reading rooms which they called *“space”,* place around females dormitories which is also known as *“Begtera”* in the students` term, dormitories, class rooms and on the field around class when teachers are late to enter the class, students` lounge, TV rooms and sometimes in cafeterias and libraries as well as on the way to class, cafeterias or libraries in the campus, and bars and restaurants, hotels and night clubs, play stations or movie houses out of the campus.

Places like dormitories, ‘Begtera’ and ‘space’ were commonly used. The place of talk depends on with whom they talk as well as the topic. A 19 years old 1st year male and female student from Nursing and Health Officer Department respectively said: *“The talk may be in the dormitory, cafeterias, at ’Begtera’, on the road, etc in the campus, and at Play stations, Bars and Restaurants, Night clubs, Hotels and etc out of the campus.” “… They communicate everything about sex and relationship at ’Begtera’.”*

Students also use these places of talk for different forms of untimely/unhealthy sexual practice which negatively influenced sexual health talk. But these places of talk could be used to initiate sexually healthy talk like through enabling female students to negotiate for safer sex, availing contraceptives to reinforce discussion on it, presenting health learning materials on different SRH topics to equip students with necessary up to date information.

#### Time (when) of talk

Time during which students met to talk on sexual issues with their peers or sex partners includes during between classes, tea-coffee ceremony arranged by the University, during reproductive health and life skill training and programs and when students` faced problem. Evening time and Saturday reported as the time for going to night clubs. Female students discussed in group about sexual issues in their dorm after one among them come back from night clubs and *“Begtera”*. Couples and sex partners took long time duration talking on sex. Students, particularly females and rural students talk mostly if they faced sexual reproductive health problem. A 23 years old 4th year female student from Electrical Engineering Department said: *“Mostly there is no planned or formal way of talking on sex and sexual health. But if someone one faced a problem there may be a talk or discussion on the problem. For example if some female student experienced pregnancy or some other problem like abortion, etc. …”*

### Partners (with whom) of talk

The FGD participants mentioned that students first and for most talk with their same sex close friends, then, with sex partner(s) and other friends. Male students talk commonly both with their male and female friends unlike female students. But female students select both the place of talk and with whom they talk. Students talk with only one close friend if it is secret things. Giving emphasis and training for female students on negotiating for safer sex and communication could help them to protect themselves and practice safe sex particularly for the talk between sex partners to make the talk healthier.

#### How students talk

Students talked sharing their experiences, opinion, ideas and knowledge. Also there was advising each other and asking for opinion from other students particularly for female students. Selecting peoples to talk with and who may keep their information confidential was common among females. The talk may be with one person or in group. The talk on sex between students was *“Hot talk”* and very attentive between couples. Male starts the talk with greeting if the talk is between opposite sexes. Some students talk to be watched by others considering their talk as their strength. Generally students who initiated sex took part of the talk voluntarily and interestingly while those who didn`t initiate sex did not participate. For example, a 23 years old 4th year male students from construction Engineering Department said: *“Sex and sexual issues are “hot issues”. But everybody is interested to talk or discuss on such issues freely. But most of the time it is not formal talk or discussion. It is expected that there are a talk or discussion in every dormitories since it is “hot issue” for everyone.*

A 19 years old 1st year female student from Nursing Department students said: *“Always it is male who starts the talk first. First they start the talk with greetings “laughing”. But if it is in the case of talk in dormitory, students in the dorm ask for her time out if she go to night club or ‘Begtera’.”* So, places of talk, when to talk and with whom to talk (or social contexts of talk) were big pillars that could negatively or positively relate to sexual health talk.

### Contents of talk

The contents of talk include: sex and sexual relationship (predominant and discussed by almost all FGDs), love and relationship (common), HIV and AIDS issues, condom and contraceptive utilization, problem occurred, pregnancy, abortion, sexually transmitted infections (STIs), cohabitation(living together), negotiating female, and previous sexual life from usually talked to less frequently talked topic. Students rarely talked about their previous sexual life, STIs other than HIV and AIDS, cohabitation. For example 21 years old 2nd year male student from Psychology Department expressed it as follow: *“Students talk on how they live together in love.… There is what we call “Cohabitation” which means how couples stay together in love or coexist and how they continue in love (or maintain their love for long period of time).”*

### Functions of talk

The functions of talk in this content mean, how the talk affects negatively (disadvantages) or positively (advantages) students` talk or how it influences the students’ sexual behavior. Advantages of talk

The advantages of talking on sex and sexual health include: experiences/information sharing, peer education, preventing/solving for problems, practicing safer sex or to know and use barriers, knowing stage of sexual intercourse, and talk for laughing/joking among others. These may in turn help students to become knowledgeable on the topic of interest. He/she may differentiate good and bad, helpful and harmful things. For example, 1st year students may have no experience and seniors may take female fresh students to unnecessary areas. So seeking an advice from wise students and talking on the issues with other students may bring the solution for many different problems. Quote from students explained the advantages of having sex and sexual health talk in preventing unintended pregnancy, having safe sexual practice, and the role it play in shaping sexual behavior of students. For example, 20 years old 2nd year female student from Psychology Department put it in the following manner: *“…Among its advantages are how to prevent unwanted pregnancy, how to have safe sexual intercourse, and how to fulfill the requirements for protected sex. Information about all things can be obtained from sharing ideas, opinions and information while talking or discussing on sex and sexual issues. So it plays roles in such a way in shaping sexual behavior of the students”.*

#### Disadvantages of talk

The common disadvantages of talk students raised include enhancement for sexual drive and motivate students to initiate or practice unsafe sex. Also following and practicing as Western people, and focusing on sexual practice than education so that it become obstacle to meet objective were another disadvantages of having the talk. Some students also said it may create conflict among students if it occurs in dormitories and may harm those students who lack awareness. The FGD discussants disclosed that the talk were mostly not educational since it focused on sexual practices. But the FGD discussants agreed up on the idea that its advantages overweight the disadvantages. A 23 years old 4th year female student from Civil Engineering Department said: *“Its disadvantage is that, those students who do not know detail about sexuality may become engaged in sexual practice as a result of hearing from friends and peers due to peer pressure. They may consider it as a good practice whenever they hear from their friends without being well informed and having readiness”.*

#### Means or channels of communication

Students used verbal and non-verbal, formal and informal communication to talk on sex and sexual health. Mobile phone call, text message and face book were the commonly used means of communications other than face-to-face communication. Telephone was the most commonly used means of communication. Also students use “sign languages” to communicate about SRH issues as a 21 years old 2nd year male student from Midwifery Department stated it below: *“Students use verbal and non-verbal communication. For example “eye blinking” shows that “he needs her for sexual relationship”. “With loud voice” they may also use other symbolic things, for example students may give the female a “flowers” or “heart shaped figures” or some other “gift”.*

Communicating through letter writing was now thought as traditional means of communication and it was replaced by telephone conversation and the later was also on the way to be replaced by social media (face book or internet). There is what students called helping relationship and they also use 3rd person as an agent to communicate on sex and male students also use what is called locally “Lekefa’ to start communication which was part of sexual harassment as it was said by 22 years old 3rd year male student from Information Technology Department. *“Most of the time… students start communication using “Lekefa”, male harassing female. Secondly, also they communicate using their friends as third party. …Then if I do have female friend and if she has also another female friends, then I ask her female friend for one of my male friend. There is also mostly studying together being male and female in “space”, in class room or in library. This is usually face-to-face communication and called helping relationship”.*

### Socio-cultural influences

A Socio-cultural influence was another theme that emerged from the data and affects students` talk negatively or positively. Male-female differences, urban-rural differences, and the influence culture in general and religion, norms and traditions in particular were some of the identified sub-theme related to socio-cultural influences.

#### Male-female differences: the influence of gender norms

Male students were free to talk on any sexual health issues including sex anywhere at any time without fear and embarrassment unlike the female students who select place, person and time of talking on any sexual health issues. Females were less talkative or silent on sexual issues but they were more exposed and affected by the problem related to sex and sexual health (see Table 2). The differences between males and females are due to culture, parenting styles, religion, gender norms and traditions. A 21years old 3rd year male student from Civics Department expressed it saying: *“There is also difference between male and female. Males do have sex talk more than females, more frequently and more freely. This may be due to culture, parenting styles, religion, gender norms, traditions and background which affects whether they have to talk or not to talk about sex and sexual health”.*

**Table 2:** Differences among male and female students concerning talking about sex and sexual health

Differences identified from FGD discussants between Male and Female Talking about Sex and Sexual Health	
Males	Females
Males don’t fear or get embarrassed talking on sex and sexual health, they talk freely	Females fear or embarrassed talking on sex and sexual health
Male talk or discuss real life situation to/with each other	Females hide things or they see as taboo talking on sex and sexual health
Male ask for love or sex	Females don’t ask male for love or sex
Male starts talk to make relationship	Females mostly don’t start sex talk or discussion on sexual issues
Males talk at anytime and anywhere	Females talk when problem occurred or when someone faced problem
It is males’ responsibility to start relationship first	Females responsible for all after the start of a relationship

If female students talked about sex and sexual health issues, they were not respected and accepted by the community and even she blamed by others as she had bad behavior, but males can talk as they likes and no one blame them as compared to counterpart. Mostly people thought ‘she was overacting” or “she was not shameful” if female talk on such an issue and also said ‘Ayenawuta’ in local Amharic term.

Starting communicating female and establishing relationship was the role of male partners while females don`t express themselves feeling early. But after relationship started, female students were responsible for any decision and selection for place and time sexual intercourses. A 20 years old 2nd year male student from History Department said: *“… early in their relationship, males start the talk using verbal communication. Females do not express their love rather they “Hide it”. But after relationship started, females would decide and tell her partner when and where to meet. She is the one who invite males for sex”.*

Males consider themselves as “hero” and they feel proud by talking on sex. The way they talk on sex and sexual issues seems that they want to be watched by others. Some females also consider themselves involved in such issues and talking on it as strength. For example the following quote is from a 25 years old 4th year male student from Electrical Engineering Department: *“They consider themselves as “hero” and they feel “proud” by talking on sex. For example, they need to be admired by others want to be said “he cheats her…”, “He uses her …”, “He makes her…” if they are males. In case of females, she wants to be supported/encouraged by others. For example, she needs to be said “she was liked by many males”. Involved in sex and sexual health talk is considered as strength of that student”.* These differences could be taken as an advantage to work on and give emphasis for female students in particular.

#### Urban-rural differences: previous residence

Talking on sex was considered as a taboo by some students especially students from rural areas and females. But students from urban areas talk freely. Students from country side fear and feel embarrassed to talk on sex and sexual health since they consider such talk as a taboo. This is influenced by culture, gender norms, religion, and previous parenting styles. Also having free talk with peers or close friends currently is the result of the presence of parent child communication during previous time.

Sex and sexual health talk between students and their families occur if they faced reproductive health problem in general and this was common among students from rural Ethiopia in particular. A 21 years old 3rd year female student from Biology Department said: *“Most of the time sex and sexual health talk between students and family takes place after the students faced reproductive health problem or if their families are free and open to talk on sexual issues. This is if they come from urban areas but students who come from rural areas fear to talk on sex and sexual health issues. So, those who come from rural Ethiopia may only talk with their families if they faced some problem”.*

Male and female students talked about the role of parents and parenting styles being conservativeness and connecting things to religion in students` sex talk and sexual health talk. Focus group discussants mentioned absence of parent child connectedness affecting students’ talk on sex and sexual health.

#### Cultural norms, religion and religious teachings

The influence of religion and other component of culture on sexuality were high and strong. There were misconceptions and dilemmas among students in higher institutions. The religious students became conservative even to talk on sex and sexual health issues. Some students were afraid even to say the word “condom” due to the influence of religion or religious beliefs. In similar way the influence of religion and religious teaching not only prevent students from having free talk or discussion but may also create inability to choose from scientifically proved and evidence based practice and traditional practices. A 22 years old 2nd year male student from Chemical Engineering Department said: *“Following what science says is difficult due to the influence of religion and beliefs. For example, in Christianity, most of the time peoples do not advised to use contraceptives. It is said to be killing children in wombs. But most of the time students even not compliant to the sayings”.*

The influence of religious teaching on contraceptive utilization including condom use was also related to the idea of abstinence only till marriage. Not only condom but also other contraceptives were not permitted to use unless you married, because they say “When you are in marriage, children are the gift of God!”

Focus group participants agreed that native cultural belief and norms influences negatively as well as positively having sex and sexual health talks. There were some students who do not talk on sex with their parents completely. Such students did not share their secret to their parents, they make sexual issues secret. Also FGD participants mentioned the influences of norm on talk and sexual behavior in that it prevents students from unsafe sexual practice. For example, quote from 22 years old 4th year male student from Civil Engineering department shows this: *“Our culture influences the sexual life of students negatively or positively. Sometimes our norms restrict students (us) from some harmful and unnecessary sexual practices if we comply with it. In such away they help in shaping sexual behaving of students…”.* Culture has also both negative and positive things. It helps and harms. It also prevents talking on issues of sex and sexual health and practicing sexual intercourse.

#### Globalization - the influences of western culture

The influence of global culture or cultural diffusion is high due to internet, face book, and films related to sex. Students were following Western cultural practice and what they see on internet, face book and what they watch from films. They want to act like what they observed. They were in the way of replacing local cultural practices with that of Whites` because they take it as modernity. A 21 years old 2nd year male student from Health Officer Department said: *“…Even their practice and acts were not what I observed previously. They changed their culture or “Habesha styles” too and engaged in “Whites` style”. Students also go to night clubs and Hotels to practice different sexual intercourse”.*

Also another FGD participant, a 20 years old 2nd year student from Nursing Department, said that: *“Globalization influences students’ sexual behavior. Many students engaged in different forms of sexual practices as the result of globalization. For example, pornography affects sexuality and sexual behavior of students. While they observe such films students may initiate sex, practice unsafe sex or addicted without knowing about contraceptives and other things well. Students may engage in sexual intercourse. Foreigners have enough knowledge on every issues and what they have to do as well as how to prevent the different consequences of sex but our community lacks knowledge”.*

## Discussion

In this study, ‘Sex and Sexual Health Talk’ was the central theme grounded in the data. Students talked with peers and sexual partners about sex more than sexual health issues. These discussions typically consisted of descriptions about their sexual relationship (how, where, and with whom) and on how they could improve negotiations with female students for sexual activities. This finding is consistent with a study conducted in England, which showed sexual health was frequently described by participants as a side issue that distracted from or diluted the details of their discussions about sexual conquest and pleasure [10].

Talking about sex is also used for entertainment creating feelings of happiness and comfort for students. Even those students who do not want to talk about sex want to hear the talk on sex between their friends. But few religious and conservative students do not want to talk about SRH issues. The common reason students raised not to have sexual health talks was feeling knowledgeable on different topics of SRH in addition to socio-cultural factors. This is in line with studies conducted in Ethiopia and England that showed there is difficulty in communicating about sexual health between youth and their parents, peer or sex partner [2, 6, 10, 12–13].

Places of talk about sex and sexual health includes “space”, “Begtera”, dormitories, students` lounge, bars and restaurants, play stations and libraries as well as along the street. This finding is consistent with peer communication and sexuality fact sheet on that showed participants prefer to talk about sexuality in a private setting, just with one other person or in a small group, and somewhere where they will not be disturbed [13]. Firstly, students talk on sex and sexual issues with their same sex close friends afterward to sex partner(s) and other friends. Male students talk commonly both with their male and female friends unlike female students. Other research also showed that depending on the individual and his or her socio-cultural contexts, communication partners can be found in the family/peer group or unrelated adult confidants; parents, brother, sister; boyfriend, girlfriend, best friend, group of friends roommates, classmates; or teacher, counselor [13].

The talk may be with one person or in group. Males feel proud, hero and popular by sex talk they have. Usually the talk on sex between students is “Hot talk”. Students were very happy and feel comfortable when talked on sex. This is in contrast with other research which showed that expressed feelings were regret, shame, disappointment; trust, curiosity; being in love, pleasure, having fun; fear, nervousness, shock, overload, aggression; and sadness, worries [13]. This may be due talk is more about sex than sexual health talk.

The presence of free talk plays an important role in shaping students` sexual behaviors. Some of the advantages are experience and information sharing, peer education, knowing risks and consequences of sexual intercourse, and knowing about and using condoms and contraceptives for safe sexual practices. This finding is consistent with the peer communication and sexuality factsheet that states healthy sexual behaviour is connected with certain aspects of peer group communication such as the communication partner or type of communication [13]. These are also important for HIV and AIDS prevention programs and supported by the different Ethiopian strategies including National Reproductive Health Strategy, TB, TB HIV and Leprosy Prevention and Control Strategic Plan among the young and the never-married [14, 15].

Females and couples prefer private settings while male students talk openly and freely without fear and embarrassment. To start communication with female and establishing relationship was the role of male partners while females don`t express their feeling early. But after relationship was established, female students were responsible for every decision. These differences were there due to cultural beliefs, traditions, parenting styles, religion, gender norms and may not apply to all students. Talking on sex is considered as a taboo by some students especially students from rural areas and females. These may be related to and influenced by culture, gender norms, religion, and parenting styles. In addition having free talk with peers or close friends currently was related to and is the result of the presence of parent child communication during previous time. Some study also showed the same [7, 16–18].

The influence of religion and other components of culture on sexuality are still strong and create misconceptions among students. Religious students were more conservative about talk on sex and sexual health issues; some students were afraid even to say condom due to the influence of religious beliefs and instructions to be abstinent until marriage. It not only prevents students from having free talk or discussion, but also creates an inability to choose between scientifically-proved and evidence-based safe sex practices and unsafe sexual practices [17, 18]. This study is not without limitations. Findings are based on data gathered from a small, non-representative sample while it’s not claimed to be generalizable to all men’s and women`s sex and sexual health talk. It is also difficult to bridge the widely acknowledged gap between what young people say they do and what they actually do.

## Conclusion

Students talked with peers and sexual partners about sex more than sexual health issues. These discussions typically consisted of descriptions about their sexual relationship (how, where, and with whom) and on how they could to improve negotiations with female students for sexual activities. The places and times of discussion about sex were mostly opportunistic. The most common reason students raised not to have sexual health talks was feeling knowledgeable on different topics of sexual health in addition to socio-cultural factors. Students discussed sex and sexual issues with their same sex close friends, sex partner(s), and other friends (classmates or dorm mates). The content, place, and time for discussions about sex were influenced by gender, social-cultural norms (eg, religion), rural vs urban living, and the occurrence of sexual health issues (eg, sexually-transmitted infections or unwanted pregnancies).

Promotion of condom use must continue with targeted and focused messages for university students. Priority should be given to designing audience-specific strategies and messages to promote discussions about sex and to encourage safe sexual practices. Primary target groups should include female and rural students, who are predisposed to risky sexual behaviour due to the tendency to have less open communication styles in their families. Communication and negotiation skills should always be a priority in training. In particular, females should be encouraged to demand condom use during every sexual encounter. Encouraging students to openly discuss sexual issues through health education methods, working with them, providing education about risk factors, and teaching communication skills are all necessary steps to reinforce behavior.

### What is known about this topic

- Young women and men usually find it easier to talk seriously and openly with their friends in many social aspects;
- Peer pressure is one of the common challenge in sexual health;
- Youths have freedom when they are away from their family.

### What this study adds

- Youths talked with peers and sexual partners about sex intercourse more than sexual health issues;
- Youths among themselves have feeling of not raised sexual health talks to be seen as knowledgeable on different topics of sexual health;
- In sexual and reproductive health programs priority should be given to designing audience-specific strategies and messages to promote discussions about sex and to encourage safe sexual practices.

## References

[1] World Population Foundation, Maastricht University IM Toolkit For Planning Sexuality Education Programs: Using Intervention Mapping in Planning School- Based Sexual and Reproductive Health and Rights (SRHR) Education Programs..

[2] Mitchell K, Wellings K (1998). Talking about sexual health-London School of Hygiene & Tropical Medicine. London Sch Hyg Trop Med..

[3] Word Bank. WHO. UNDP. UNFPA (2006). Defining sexual health Report of a technical consultation on sexual health 28–31 January 2002, Geneva..

[4] MOH, HAPCO A, EPHA C, Two R (2005). HIV/AIDS Behavioral Surveillance Survey (BSS) Ethiopia 2005 Round Two HIV/AIDS Behavioral Surveillance Survey (BSS) Ethiopia 2005 Round Two..

[5] USAIDS (1999). Sexual behavioural change for HIV?: Where have theories taken us??..

[6] Taffa N, Haimanot R, Desalegn S, Tesfaye A, Kedir M Do parents and young people communicate on sexual matters'?. The situation of Family Life Education (FLE) in a rural town in Ethiopia..

[7] Alicia W (2010). The Facts Parent-Child Communican: Promoting Sexually health Youth. Advocates for Youth Right.Respect.Responsibility]..

[8] Talking to your partner about sex [Internet]..

[9] Assebe T Assessment of Factors Affecting Parents in Discussing Reproductive Health Issue with their Adolescent in Shambo town, Horo Guduru Wollega, Oromiya Region, Ethiopia, May 200 A. 200..

[10] Knight R, Shoveller JA, Oliffe JL, Gilbert M, Frank B, Ogilvie G (2012). Masculinities, “ guy talk ” and “ manning up ”: a discourse analysis of how young men talk about sexual health. Sociol Health Illn..

[11] Debre Birhan University Debre Berhan University Annual Magazine..

[12] GebreYesus D, Fantahun M (2010). Assessing communication on sexual and reproductive health issues among high school students with their parents, Bullen Woreda, Benishangul Gumuz Region, North West Ethiopia. Ethiop J Heal Dev..

[13] IPPF EN (2012). Peer communication and sexuality factsheet on qualitative research [Internet]..

[14] Ambaw F, Mossie A, Gobena T (2010). Sexual Practices and Their Development Pattern among Jimma University Students. Ethiop J Heal Sci..

[15] FMOH Ethiopia (2007). Tuberclosis, TB/HIV and Leprosy Prevention and Control Stratic Plan, 2007/8 – 2009/10..

[16] Health MOF (2006). National Reproductive Health Strategy, 2006-2015..

[17] Ation P (2000). Issues at a Glance: Parent-Child Communication Programs Helping Parents Become Knowledgeable and Comfortable as Sex Educators Research (Internet)..

[18] Holtzman D, Rubinson R (2013). Parent and peer communication effects on AIDS-related behavior among US high school students. Fam Plann Perspect..

